# Risk factors for surgical site infections in abdominal surgeries in Ghana: emphasis on the impact of operating rooms door openings

**DOI:** 10.1017/S0950268820001454

**Published:** 2020-07-01

**Authors:** A. A. A. Bediako-Bowan, K. Mølbak, J. A. L. Kurtzhals, E. Owusu, S. Debrah, M. J. Newman

**Affiliations:** 1 Department of Surgery, University of Ghana Medical School, University of Ghana, Accra, Ghana; 2 Department of Surgery, Korle Bu Teaching Hospital, Accra, Ghana; 3 Department of Veterinary and Animal Science, University of Copenhagen, Copenhagen, Denmark; 4 Division of Infectious Disease Preparedness, Statens Serum Institut, Copenhagen, Denmark; 5 Centre for Medical Parasitology at Department of Immunology and Microbiology, University of Copenhagen, Copenhagen, Denmark; 6 Department of Clinical Microbiology, Copenhagen University Hospital (Rigshospitalet), Copenhagen, Denmark; 7 Department of Medical Laboratory Sciences, School of Biomedical and Allied Health Science, University of Ghana, Accra, Ghana; 8 Department of Surgery, University of Cape Coast, Cape Coast, Ghana; 9 Department of Medical Microbiology, University of Ghana Medical School, University of Ghana, Accra, Ghana

**Keywords:** Epidemiology, Ghana, risk factors, surgical site infections

## Abstract

Major surgery carried out in low- and middle-income countries is associated with a high risk of surgical site infections (SSI), but knowledge is limited regarding contributory factors to such infections. This study explores factors related to patients developing an SSI in a teaching hospital in Ghana. A prospective cohort study of patients undergoing abdominal surgical procedures was conducted at Korle Bu Teaching Hospital. Patient characteristics, procedures and environmental characteristics were recorded. A 30-day daily surveillance was used to diagnose SSI, and Poisson regression analysis was used to test for association of SSI and risk factors; survival was determined by proportional hazard regression methods. We included 358 patients of which 58 (16.2%; 95% CI 12.7–20.4%) developed an SSI. The median number of door openings during an operation was 79, with 81% being unnecessary. Door openings greater than 100 during an operation (*P* = 0.028) significantly increased a patient's risk of developing an SSI. Such patients tended to have an elevated mortality risk (hazard ratio 2.67; 95% CI 0.75–9.45, *P* = 0.128). We conclude that changing behaviour and practices in operating rooms is a key strategy to reduce SSI risk.

## Introduction

Major surgery carried out in low- and middle-income countries (LMIC) is associated with a high risk of surgical site infections (SSI), with an increased burden in terms of morbidity, mortality and costs for patients [1]. Multiple risk factors for SSI have been identified [2] and the World Health Organisation (WHO) has published recommendations for their prevention [3]. However, the relative contribution of these risk factors in LMIC is almost unknown.

Intervention studies with the aim to reduce SSI in sub-Saharan Africa have been largely based on strategies developed in high-income countries [4]. Other limitations in the existing body of evidence include variations in, amongst others, SSI definitions and follow-up periods [5]. The limited knowledge of the factors related to SSI in LMIC makes it challenging to target strategies to reduce infection rates due to cost implications of interventions for poorly resourced countries. Thus, knowledge of the risk factors that impact in such countries will help to inform cost-effective interventions aimed at reducing SSI. This study was designed to explore the relative contribution of factors associated with the risk of SSI in patients undergoing abdominal surgery in a teaching hospital in such a setting.

## Methods

Data were collected at the general surgery unit at the Korle Bu Teaching Hospital (KBTH), a tertiary hospital in Ghana. The theatre complex for general surgery comprises seven operating rooms (OR) of similar size and architecture, each with four doorways (apart from one with a single doorway) and equipped with a non-laminar flow ventilation system set between 15 and 25 °C. The hospital is involved in the training of residents, medical students, nursing students and allied health practitioners, all spending some learning hours in the OR.

Skin preparation solution, ‘Savlon in spirit’ (10% Savlon (0.15% chlorhexidine, 1.5% cetrimide and water) and 34% methylated spirit (95% ethanol and 5% methanol)), is usually prepared in house but other skin preparation solutions are purchased commercially. Antibiotic prophylaxis in the department is based on general principles according to national standard treatment guidelines [6].

### Study design

We conducted a prospective cohort study of all patients undergoing abdominal surgical procedures at the general surgery unit between the hours of 8:00 and 21:00 from 1 February to 31 July 2019. Patients undergoing implant surgery or whose wounds were not primarily closed in the OR were excluded. The study formed part of ongoing surveillance of SSI but with the collection of additional exposure variables [7]. Ethical approval was granted by the Korle Bu Teaching Hospital Institutional Review Board, KBTH-STC/IRB/00005/2019 and the College of Health Sciences' Ethical and Protocol review committee, CHS-Et/M.5 – 4.10/2018-2019. All patients gave written informed consent.

### Procedure

Information on patient characteristics including age, sex, American Society of Anaesthesiologist (ASA) physical status classification [8], co-morbidities (previous diagnosis of diabetes, HIV, sickle cell disease, presence of a malignancy or tuberculosis), use of substances such as alcohol, cigarettes or drugs was recorded on a structured questionnaire. Weight and height were measured to calculate body mass index. Surgical procedure characteristics were classed according to urgency, wound contamination/class [8], type of operation, surgeon's experience duration of procedure (from time of incision to wound closure) and peri-operative use of antibiotics.

The following environmental characteristics were recorded based on direct observation: presence or not of running water, functioning ventilation system, power cuts during the procedure, use of several instrument sets for a procedure, the number of persons present in an OR (excluding the patient and the researcher, monitored at 20 min intervals) and the number of door openings during a procedure [9]. Reasons for door openings were grouped according to needs to finish the procedure or secure the patients' safety.

A 30-day surveillance period involving daily in-patient assessment and post-discharge surveillance as described in a previous study [7] was used to diagnose SSI. Furthermore, outcomes such as the need for additional radiological or surgical intervention, development of other hospital-acquired infections and mortality during the 30-day follow-up were recorded.

### Statistical analysis

Poisson regression using the binomial model was used to test for association of SSI and risk factors, through relative risks ratios. Univariable analysis of patient, procedure and environmental characteristics was conducted and variables with *P* < 0.1 were entered into a multivariable model for analysis. Cox regression (proportional hazard regression analysis) was used to estimate the relative risk of mortality. We applied the development of an SSI as a time-dependent covariate. Statistical analyses were performed using Stata/MP version 15.1 (Stata Corp., College Station, TX, USA).

## Results

**Fig. 1.**
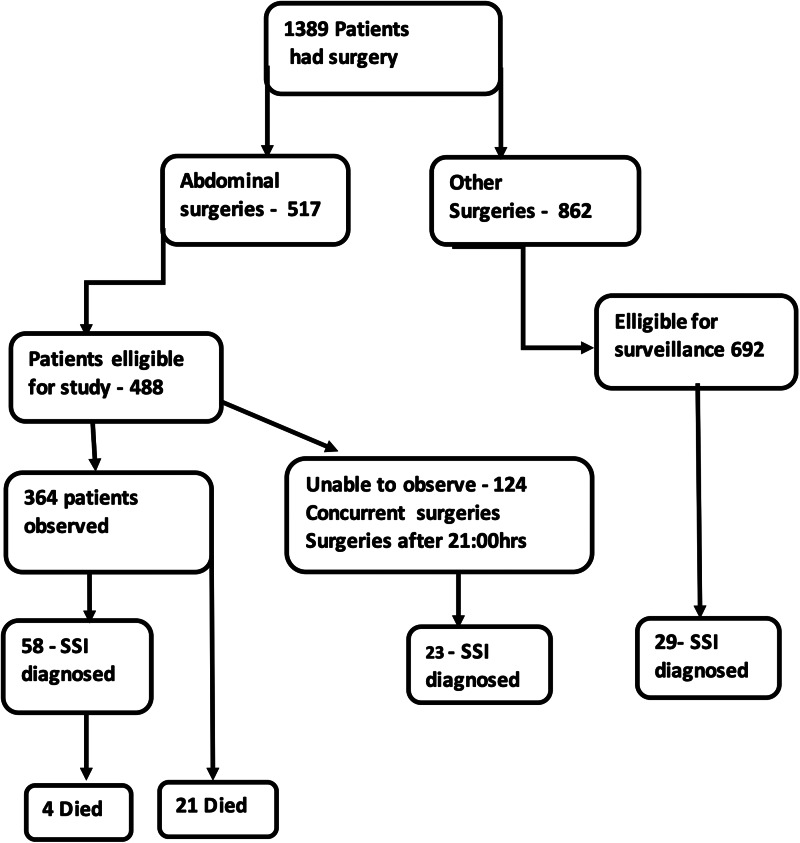
Flow chart of patients undergoing surgery in the general surgery unit of the teaching hospital showing the number undergoing abdominal surgeries or other surgeries – the number of abdominal surgeries eligible for the study and the actual number involved in the study to describe the risk factors of SSI.

In all, 517 abdominal surgeries were carried out over the period and 488 were deemed eligible for the study; of these, 364 (75%) were observed over the period (Fig. 1). We excluded data from seven patients (three were transferred out of the general surgery unit and four were entered twice due to a second surgical procedure for an SSI). Fifty-eight of the 358 included patients (16.2%; 95% confidence interval (CI) 12.7–20.4%) developed an SSI, 37 (63.8%) in the inpatient setting and 21 (36.2%) post-discharge.

### Patient characteristics

The male:female ratio was 1:1, with a median age of 41years (interquartile range (IQR) 27–56 years). They had a median body mass index of 24.6 (IQR 21.4–28.0). In all, 113 patients had co-morbidities including 27 (7.5%) with diabetes, 90 (25.1%) with a malignancy, of whom 12 (3%) had received chemotherapy within 12 weeks before surgery. Patients with sickle cell disease were of SS- (5/6) or SC-genotype (1/6).

### Procedure characteristics

Fifty-two per cent (185/358) of procedures were emergencies and 3% (11/358) were re-operations. Antibiotic therapy was instituted in 145 (40%) patients within a day (IQR 1–2 days) before surgery, 81% of whom had wounds classified as contaminated or dirty, 14% as clean contaminated and 5% as clean. A total of 338 (94.4%) patients received prophylactic antibiotic therapy: 64% (23/36) of patients with clean wounds, 93% (88/95) with clean contaminated and all patients with contaminated (151) or dirty wounds (76). Prophylactic antibiotics were started within an hour before surgical incision in 82% (277/338) of patients but after the incision was made in 18% (61/338).

All, but 12, patients (326/338) had antibiotics continued after surgery for a median of 5 days (IQR 2–7 days): 40 patients for a day, 166 for 1–5 days and 120 for more than 5 days. For patients who had antibiotics continued for 1–5days, 71% (118/166) was for treatment of contaminated and dirty wounds and 29% (48/166) for clean and clean contaminated wounds. For antibiotics given for more than 5 days, 83% (101/121) was for treatment of contaminated and dirty wounds, and 17% (20/121) for clean and clean contaminated. Overall, 55% (68/118) of patients with clean or clean contaminated wounds had antibiotics continued for more than a day post-operatively.

### Environmental characteristics

In seven (2%) procedures, mains tap water was unavailable and water stored in containers was used. Power cuts, usually intermittent and of short duration, occurred during 16 (4.5%) procedures and an automated standby generator was used. Likewise, malfunction of the ventilation system occurred during 12 (3.3%) procedures, and was linked to power cuts. During 222 (62%) procedures, extra instrument sets were brought into the OR as required.

A median of 8 (IQR 7–10, range 3–19) persons were present in the OR at any given time and a total of 32 684 door openings was recorded, giving on average 91 occasions during 358 procedures, the great majority (81%) of which were considered unnecessary (Table 1). Elective surgery showed more door openings, 117 (median 56, IQR 34–92) compared to 72 (median 100, IQR 73–146) for emergency surgery; bowel surgery accounted for 55% of the procedures in which there were >100 door openings. Additional patient, procedure and environmental characteristics are summarised in Supplementary Table S1.

**Table 1.** Reasons for 32 684 door openings during 358 operations

Necessary door openings			Semi-necessary door openings			Unnecessary door openings		
Reasons	Sum	% of total	Reasons	Sum	% of total	Reasons	Sum	% of total
Expert consultations	209	0.6	Surgical team members	601	1.8	Logistics unrelated to procedure	4409	13.5
Instruments and materials needed for operation	5356	16.4	Lunch break	35	0.1	Students moving in and out	2049	6.3
						Social visits	7428	22.7
						Undetectable reasons	12 597	38.5
Total	5565	17.0		636	1.9		26 483	81.0

### Clinical outcomes

Twelve patients who developed an SSI within 30 days of surgery had another radiological (2) or surgical intervention (10). Twenty-nine patients developed other hospital-acquired infections, including pneumonia (12), urinary tract infection (7), blood stream infection (4) and others (6); 11 of these patients also had an SSI. Patients who developed an SSI had a 7% (4/58) mortality risk, similar for those who did not (7%; (21/300)). The relative mortality risk for patients with an SSI was however 2.67 (CI 0.75–9.45; *P* = 0.128), adjusting for the time at risk, age, the presence of comorbidity and another hospital-acquired infection. The relative mortality risk for patients with hospital-acquired infections (including SSI) was 4.56 (2.18–9.51; *P* = 0.001), when adjusted for age and comorbidity.

### Analysis of risk factors for SSI

Overweight patients had double the risk of developing an SSI (risk ratio (RR) 2.1, 95% CI 1.11–3.98) than patients with normal weight in univariable analysis. The presence of a comorbidity tended to increase a patient's risk of developing an SSI (1.53, 0.92–2.58), especially for diabetics (1.68, 0.76–3.71) and for patients who had undergone chemotherapy within the last 12 weeks prior to surgery (1.57, 0.49–5.02).

Bowel surgery, including appendicectomy had a three times increased risk for an SSI (3.11, 0.75–12.85) compared to ventral hernia repairs, and procedures lasting 60–120 and >120 min had an approximate twofold higher risk of SSI. Wound class was significantly associated with SSI incidence risk, increasing with increasing levels of contamination (Table 2).

**Table 2.** Patient factors related to surgical site infections

Characteristics	*n* (%)	Number with SSI	Incidence risk of SSI (number of infections per 100 procedures) (95% CI)	Univariable analysis Incidence risk ratio (95% CI)	*P*-value
Comorbidity					
No	245	34	13.9 (9.8–18.8)	–	0.116
Yes	113	24	21.2 (14.1–29.9)	1.53 (0.91–2.58)	
Wound class					
Clean	36 (10.1)	3	8.3 (1.8–22.5)	–	0.049
Clean contaminated	95 (26.5)	10	10.5 (5.2–18.5)	1.26 (0.35–4.58)	
Contaminated	151 (42.2)	25	16.6 (11.0–23.5)	1.99 (0.60–6.58)	
Dirty	76 (21.2)	20	26.3 (16.8–37.7)	3.15 (0.94–10.62)	
Duration of surgery					
*Median (IQR)	80 (48–120) min				
0–60 min	123 (34.4)	10	8.1 (4.0–14.4)	–	0.015
60–120 min	144 (40.2)	29	20.1 (13.9–27.9)	2.48 (1.21–5.08)	
>120 min	91 (25.4)	19	20.9 (13.1–30.7)	2.57 (1.19–5.52)	
Door openings per procedure					
*Median (IQR)	79 (44–115)				
<100	234 (65.4)	25	10.6 (7.0–15.4)	–	0.001
>100	124 (34.6)	33	26.6 (19.1–35.3)	2.49 (1.48–4.18)	

CI, confidence interval; *Median (IQR), median with interquartile range of variable.

*P*-value >0.05 was considered significant. *P*-values are based on the likelihood ratios.

Operations with >100 door openings more than doubled the risk of developing an SSI (2.49, 1.48–4.18) (Table 2), and the presence of >10 persons in the OR tripled this risk (3.12, 0.71–13.66), compared with procedures with <5 persons in the OR. After an age- and comorbidity-adjusted multivariable analysis of factors with *P* < 0.1, the number of door openings remained significantly associated with the RR for developing an SSI (2.25, 1.09–4.66; *P* = 0.028).

## Discussion

We explored multiple factors associated with SSI after abdominal surgery in a surgical department in Ghana. The 30-day SSI incidence risk was 16% and as high as 20% after bowel surgery. This observation corroborates that SSI remains a common hospital-acquired infection in LMIC, with a risk similar to the 14% pooled SSI risk for middle-income countries [10]. Our key finding was that increased door openings during a procedure significantly contributed to the increased risk of developing an SSI which is in line with our previous observations on the importance of traffic flow and microbial air contamination in ORs [9, 11].

Door openings have been associated with increased microbial air contamination in OR [9, 12, 13]. Reducing the number of persons in the room and enforcing behavioural changes such as disallowing the opening of doors during surgery are cost-effective strategies to help reduce SSI. Being a teaching hospital with persons under training, the tendency for staff and students to walk in and out of the OR during a procedure is high, as shown in this study, and this is rarely done for the purpose of completing the procedure or securing patient safety. Unnecessary social visits as well as staff walking in and out of the OR for no apparent reason during an operation should be actively discouraged. Proper planning by staff and a central logistic distributing area within the theatre complex may reduce the need for staff to enter functioning ORs for logistics for other ongoing operations. Even though door openings for the purpose of bringing in logistics related to an operation were classified as necessary, proper planning by staff may also reduce these. Giving priority to the patient outweighs the need for teaching and training. Thus, other teaching and training methods must be found that do not increase SSI risk and, when students are present in an OR, they should remain in the room throughout the procedure.

Due to lack of resources, staff tend to open several instrument sets to find the needed instruments for procedures. Though this feature may also be a proxy for the number of door openings, our study suggests an increased risk for SSI for procedures where several instrument sets are opened, although with a wide confidence interval in the multivariable model. This may either be due to the lack of planning by the OR staff or lack of resources to adequately prepare complete instrument sets. Resourcing operating theatres in LMIC with the necessary number of instruments affords the staff to open single equipment sets with all that is required for a procedure and help reduce the risk of SSI. Larger studies are required to determine how many sets can be opened without compromising patient safety.

Several SSI interventional studies relating to antibiotic use in surgical practice have been performed in LMIC [5, 14, 15], and prolonging antibiotic therapy beyond the time needed for prophylaxis remains a challenge in surgical departments in Ghana [16]. We found that approximately one-third of the patients who had antibiotics continued for 1–5 days, and almost a fifth of those continued beyond 5 days, had clean or clean contaminated wounds. There is a need for increased education on the appropriate use of antibiotics in surgical practice to reduce the problem of antimicrobial resistance. Almost 20% of prophylactic antibiotics were administered after skin incision in this study and this has been associated with higher SSI incidence. Timing of prophylactic antibiotic use in the OR can be improved by adhering to the recommended WHO surgical safety checklist [17].

The present study was not sufficiently powered to explore patient- and procedure-related risk factors in a comprehensive manner with a relatively small number of subjects. Nonetheless, the findings corroborate the importance of factors such as wound class, duration of procedure, overweight and underlying illness, although there is a large uncertainty around our point estimates. Wound class and the duration of a procedure remain some of the readily recognised risk factors associated with SSI, although duration itself may not be an independent risk factor but rather a reflection of human traffic during the procedure. Hence long duration was not identified in the multivariable model as a risk factor.

Comorbidity, particularly diabetes, increases the risk of an SSI but further studies are warranted to describe the SSI risk with other comorbidities such as sickle cell disease, HIV and tuberculosis in LMIC. As our hospital is a tertiary referral centre, there are higher numbers of patients with malignancies or those on chemotherapy undergoing surgery and further studies will help describe their risk for developing an SSI.

Overweight and obesity is on the rise in Ghana [15]. We reiterate the recommendation that for non-emergency procedures, patients are asked to reduce their weight before surgery to reduce their risk of SSI. The low smoking prevalence in Ghana is reassuring [18], but due to its association with surgical complications, we would still routinely recommend smoking cessation before elective surgery [19].

In conclusion, our study strongly suggests that interventions aimed at reducing the numbers of personnel, and restricting their movement in and out of the OR during surgery, will have a great impact on the risk of SSI in LMIC. Such measures are simple and feasible and likely to be cost-effective. The study also corroborates the importance of patient- and procedure-related factors, albeit with more uncertainty.

## Data Availability

Data are not publicly available for ethical reasons but are available from the authors upon request.

## Supplementary material

For supplementary material accompanying this paper visit http://dx.doi.org/10.1017/S0950268820001454. click here to view supplementary material
