# Supplementary material for: Risk factors for surgical site infections in abdominal surgeries in Ghana: emphasis on the impact of operating rooms door openings
Source: Epidemiol Infect. 2020 Jul 1;148:e147. doi: 10.1017/S0950268820001454 (PMC7398855; doi:10.1017/S0950268820001454)
Supplement: Supplementary file 1 [file S0950268820001454sup001.docx]

*‘Epidemiology and Infection’* Risk factors for surgical site infections in abdominal surgeries in Ghana: emphasis on the impact of operating rooms door openings

A. A. A. BEDIAKO-BOWAN^1, 2, 3, 4^, K. MØLBAK^3, 4^, J. A. L. KURTZHALS^5, 6^, E. OWUSU^7^, S. DEBRAH^8^, M. J. NEWMAN^9^.

‘SUPPLEMENTARY MATERIAL’

Supplementary Table S1: Patient factors related to surgical site infections

| Characteristics | n (%) | Number with SSI | Incidence risk of SSI (number of infections per 100 procedures)  (95% CI) | Univariable analysis | | Multivariable analysis | |
| --- | --- | --- | --- | --- | --- | --- | --- |
|  |  |  |  | Incidence risk ratio (95% CI) | P-value | Incidence risk ratio (95% CI) | P-value |
| Patient characteristics | | | | | | | |
| Gender | | | | | | | |
| Male | 183 (51.1) | 30 | 16.3 (11.3-22.6) | - | 0.926 |  |  |
| Female | 175 (48.9) | 28 | 16.0 (10.9-22.2) | 0.98 (0.58 – 1.63) |  |  |  |
| Age | | | | | | | |
| *Median (IQR) | 41 (27; 56) years |  |  |  |  |  |  |
| 10-29 | 106 (29.6) | 14 | 13.2 (7.4-21.1) | - | 0.716 |  | 0.641 |
| 30-49 | 124 (34.6) | 23 | 18.5 (12.1-26.5) | 1.40 (0.72 – 2.37) |  | 1.45 (0.71-2.95) |  |
| 50-69 | 97 (27.1) | 17 | 17.5 (10.5-26.5) | 1.32 (0.65 – 2.69 ) |  | 1.13 (0.49-2.61) |  |
| >70 | 31 (8.7) | 4 | 12.9 (3.6-29.8) | 0.98 (0.32 – 2.97) |  | 0.85 (0.26-2.77) |  |
| BMI | | | | | | | |
| *Median (IQR) | 24.61 (21.4; 28.0) |  |  |  |  |  |  |
| Normal weight | 141 (39.4) | 17 | 12.0 (7.2 – 18.6) | - | 0.199 |  |  |
| Underweight | 18 (5.0) | 2 | 11.1 (1.4-34.7) | 0.92 (0.21 – 3.99) |  |  |  |
| Overweight | 83 (23.2) | 21 | 25.3 (16.4-36.3) | 2.10 (1.11 – 3.98) |  |  |  |
| Obese | 56 (15.6) | 10 | 17.9 (8.9-30.4) | 1.48 (0.68 – 3.23) |  |  |  |
| Missing | 60 (16.8) | 8 | 13.3 (5.9 – 24.6) | 1.11 (0.48 – 2.56) |  |  |  |
| ASA | | | | | | | |
| Class I | 150 (41.9) | 18 | 12.0 (7.3-18.3) | - | 0.254 |  |  |
| Class II | 122 (34.1) | 23 | 18.9 (12.3-26.9) | 1.57 (0.85 – 2.91) |  |  |  |
| Class III | 78 (21.8) | 14 | 17.9 (10.2-28.3) | 1.49 (0.74 – 3.00) |  |  |  |
| Class IV | 8 (2.2) | 3 | 37.5 (8.5-75.5) | 3.12 (0.92 – 10.60) |  |  |  |
| Comorbidity | | | | | | | |
| No | 245 | 34 | 13.9 (9.8-18.8) | - | 0.116 |  | 0.181 |
| Yes | 113 | 24 | 21.2 (14.1-29.9) | 1.53 (0.91 – 2.58) |  | 1.53 (0.82-2.84) |  |
|  |  |  |  |  |  |  |  |
| Diabetes |  |  |  |  |  |  |  |
| No | 331 (92.5) | 51 | 15.4 (11.7-19.7) |  | 0.227 |  |  |
| Yes | 27 (7.5) | 7 | 25.9 (11.1-46.3) | 1.68 (0.76 – 3.71) |  |  |  |
|  |  |  |  |  |  |  |  |
| HIV |  |  |  |  |  |  |  |
| No | 355 (99.2) | 58 | 16.3 (12.6-20.6) | - | 0.323 |  |  |
| Yes | 3 (0.8) | 0 | 0 | 0.00 (0.00 – 0.00) |  |  |  |
|  |  |  |  |  |  |  |  |
| Tuberculosis |  |  |  |  |  |  |  |
| No | 354 (98.9) | 58 | 16.4 (12.7-20.6) | - | 0.254 |  |  |
| Yes | 4 (1.1) | 0 | 0 | 0.00 (0.00 – 0.00) |  |  |  |
|  |  |  |  |  |  |  |  |
| Sickle cell disease |  |  |  |  |  |  |  |
| No | 352 (98.3) | 58 | 16.5 (12.8-20.8) | - | 0.161 |  |  |
| Yes | 6 (1.7) | 0 | 0 | 0.00 (0.00 – 0.00) |  |  |  |
|  |  |  |  |  |  |  |  |
| Chemotherapy |  |  |  |  |  |  |  |
| No | 346 (96.6) | 55 | 15.9 (12.2-20.2) | - | 0.475 |  |  |
| Yes | 12 (3.4) | 3 | 25 (5.5-57.2) | 1.57 (0.49 – 5.02) |  |  |  |
| Malignancy present |  |  |  |  |  |  |  |
| No | 268 (74.9) | 40 | 14.9 (10.9-19.8) |  | 0.312 |  |  |
| Yes | 90 (25.1) | 18 | 20.0 (12.3-29.7) | 1.34 (0.77 – 2.34) |  |  |  |
|  |  |  |  |  |  |  |  |
| Substance use | | | | | | | |
| No | 282 (78.8) | 50 | 17.7 (13.4-22.7) |  | 0.145 |  |  |
| Yes | 76 (21.2) | 8 | 10.5 (4.6-19.7) | 0.59 (0.28 – 1.25) |  |  |  |
| Drug abuse |  |  |  |  |  |  |  |
| No | 349 (97.5) | 56 | 16.0 (12.3-20.3) |  | 0.667 |  |  |
| Yes | 9 (2.5) | 2 | 22.2 (2.8-60.0) | 1.38 (0.34 – 5.27) |  |  |  |
|  |  |  |  |  |  |  |  |
| Alcohol use |  |  |  |  |  |  |  |
| No | 301 (84.1) | 53 | 17.6 (13.5-22.4) | - | 0.100 |  |  |
| Yes | 57 (15.9) | 5 | 8.8 (2.9-19.2) | 0.50 (0.20 – 1.25) |  |  |  |
|  |  |  |  |  |  |  |  |
| Smoking status |  |  |  |  |  |  |  |
| Never smoked | 316 (88.3) | 53 | 16.8 (12.8-21.3) | - | 0.245 |  |  |
| Previous smoker | 22 (6.1) | 1 | 4.5 (0.1-22.8) | 0.27 (0.04 – 1.93) |  |  |  |
| Current smoker | 20 (5.6) | 4 | 20.0 (5.7-43.7) | 1.19 (0.41 – 3.29) |  |  |  |
| Procedure characteristics | | | | | | | |
| Urgency of procedure | | | | | | | |
| Elective | 17348.3) | 29 | 16.8 (11.5-23.2) | - | 0.799 |  |  |
| Emergency | 185 (51.7) | 29 | 15.7 (10.8-21.7) | 0.94 (0.56 – 1.56) |  |  |  |
| Surgeon type | | | | | | | |
| Junior resident | 84 (23.5) | 14 | 16.7 (9.4-26.3) | - | 0.462 |  |  |
| Senior resident | 100 (27.9) | 13 | 13.0 (7.1-21.2) | 0.78 (0.36 – 1.66) |  |  |  |
| Consultant | 174 (48.6) | 31 | 17.8 (12.4-24.3) | 1.07 (0.57 – 2.01) |  |  |  |
| Procedure performed | | | | | | | |
| Ventral hernia repair | 31 (8.7) | 2 | 6.4 (0.8-21.4) | - | 0.166 |  |  |
| Bowel surgery | 218 (60.9) | 44 | 20.2 (15.1-26.1) | 3.13 (0.76 – 12.90) |  |  |  |
| Biliary, liver, pancreas surgery | 52 (14.5) | 6 | 11.5 (4.3-23.4) | 1.79 (0.36– 8.86) |  |  |  |
| Gastric surgery | 22 (6.1) | 2 | 9.1 (1.1-29.2) | 1.40 (0.20 – 10.00) |  |  |  |
| Exp. Laparotomy + other abdominal surgery | 35 (9.8) | 4 | 11.4 (3.2-26.7) | 1.77 (0.32 – 9.67) |  |  |  |
| Operation type | | | | | | | |
| Primary operation | 347 (96.9) | 55 | 15.9 (12.1-20.1) | - | 0.398 |  |  |
| Re-operation | 11 (3.1) | 3 | 27.3 (6.0-61.0) | 1.72 (0.54 – 5.50) |  |  |  |
| Antibiotics used before surgery | | | | | | | |
| No | 213 (59.5) | 33 | 15.5 (10.9-21.1) | - | 0.688 |  |  |
| Yes | 145 (40.5) | 25 | 17.2 (11.5-24.4) | 1.11 (0.66– 1.87) |  |  |  |
| Antibiotics for prophylaxis | | | | | | | |
| No | 20 (5.6) | 2 | 10.0 (1.2-31.7) | - | 0.447 |  |  |
| Yes | 338 (94.4) | 56 | 16.6 (12.8-21.0) | 1.66 (0.40 – 6.79) |  |  |  |
| Antibiotics continued after surgery | | | | | | | |
| No | 32 (8.9) | 2 | 6.3 (0.7-20.8) | - | 0.097 |  | 0.910 |
| Yes | 326 (91.1) | 56 | 17.2 (13.2-21.7) | 2.75 (0.67 – 11.26) |  | 1.13 (0.14-9.09) |  |
| Wound class | | | | | | | |
| Clean | 36 (10.1) | 3 | 8.3 (1.8-22.5) | - | 0.049 |  | 0.142 |
| Clean contaminated | 95 (26.5) | 10 | 10.5 (5.2-18.5) | 1.26 (0.35 – 4.58) |  | 0.88 (0.17-4.03) |  |
| Contaminated | 151 (42.2) | 25 | 16.6 (11.0-23.5) | 1.99 (0.60 – 6.58) |  | 1.47 (0.32-6.73) |  |
| Dirty | 76 (21.2) | 20 | 26.3 (16.8-37.7) | 3.15 (0.94 – 10.62) |  | 2.20 (0.47-10.35) |  |
| Duration of surgery | | | | | | | |
| *Median (IQR) | 80 (48 – 120) minutes |  |  |  |  |  |  |
| 0-60 minutes | 123 (34.4) | 10 | 8.1 (4.0-14.4) | - | 0.015 |  | 0.661 |
| 60-120 minutes | 144 (40.2) | 29 | 20.1 (13.9-27.6) | 2.48 (1.21 – 5.08) |  | 1.18 (0.50-2.77) |  |
| >120 minutes | 91 (25.4) | 19 | 20.9 (13.1-30.7) | 2.57 (1.19 – 5.52) |  | 0.87 (0.30-2.56) |  |
| Environmental characteristics | | | | | | | |
| Ventilation system functioning | | | | | | | |
| Yes | 346 (96.6) | 57 | 16.5 (12.7-20.9) | - | 0.449 |  |  |
| No | 12 (3.4) | 1 | 8.3 (0.2-38.5) | 0.51 (0.07 – 3.65) |  |  |  |
| Power cuts | | | | | | | |
| No | 342 (95.5) | 55 | 16.18 (12.3-20.4) | - | 0.800 |  |  |
| Yes | 16 (4.5) | 3 | 18.8 (4.5-45.6) | 1.17 (0.36 – 3.72) |  |  |  |
| Skin preparation solution | | | | | | | |
| Povidone iodine | 59 (16.5) | 3 | 18.6 (9.7-30.9) | - | 0.709 |  |  |
| Povidone iodine + alcohol | 17 (4.7) | 3 | 17.6 (3.8-43.4) | 0.50 (0.16 – 1.62) |  |  |  |
| Chlorhexidine + alcohol | 10 (2.8) | 41 | 30.0 (6.7-65.2) | 0.59 (0.12 – 2.91) |  |  |  |
| Savlon in spirit | 272 (76.0) | 11 | 15.1 (11.0-19.9) | 0.60 (0.17 – 2.22) |  |  |  |
| Running water | | | | | | | |
| Yes | 351 (98.0) | 56 | 22.2 (12.2-20.2) | - | 0.458 |  |  |
| No | 7 (2.0) | 2 | 28.6 (3.7-71.0) | 1.79 (0.44 – 7.34) |  |  |  |
| Extra instrument sets opened | | | | | | | |
| No | 136 (38.0) | 15 | 11.0 (6.3-17.5) | - | 0.050 | 1.28 (0.66-2.48) | 0.450 |
| Yes | 222 (62.0) | 43 | 19.4 (14.3-25.2) | 1.76 (0.98 – 3.16) |  |  |  |
| Persons present per procedure | | | | | | | |
| *Median (IQR) | 8 (7 – 10) |  |  |  |  |  |  |
| 0-5 | 25 (7.0) | 2 | 8.0 (1.0-26.0) |  | 0.137 |  |  |
| 5-10 | 273 (76.3) | 41 | 15.0 (10.1-19.8) | 1.88 (0.45 – 7.76) |  |  |  |
| >10 | 60 (16.8 | 15 | 25.0 (14.7-37.9) | 3.12 (0.71 – 13.66) |  |  |  |
| Door openings per procedure | | | | | | | |
| *median (IQR) | 79 (44 – 115) |  |  |  |  |  |  |
| <100 | 234 (65.4) | 25 | 10.6 (7.0-15.4) | - | 0.001 | 2.25 (1.09-4.66) | 0.028 |
| >100 | 124 (34.6) | 33 | 26.6 (19.1-35.3) | 2.49 (1.48 – 4.18) |  |  |  |

CI - Confidence interval, *Median (IQR) – median with interquartile range of variable. p-value >0.05 was considered significant. P-values are based on the likelihood ratios. Variables with p<0.1 were entered into a multivariable model and adjusted for age and comorbidity. Exp - exploratory
